# Annotated drowsiness detection dataset captured using Raspberry Pi 5

**DOI:** 10.1016/j.dib.2025.112211

**Published:** 2025-10-21

**Authors:** Suryadiputra Liawatimena, Nugro Isworo

**Affiliations:** a Computer Science Department, BINUS Graduate Program - Master of Computer Science, Bina Nusantara University, Jakarta, 11480, Indonesia; b Automotive & Robotics Program, Computer Engineering Department, BINUS ASO School of Engineering, Bina Nusantara University, Jakarta, 11480, Indonesia

**Keywords:** Drowsiness detection, Edge computing, Raspberry Pi, Computer vision, Automotive safety, FOMO algorithm, Real-time processing

## Abstract

Drowsiness-related accidents represent a critical safety concern in transportation and workplace environments, necessitating real-time monitoring solutions deployable on affordable hardware. This paper introduces the Annotated Drowsiness Detection Dataset, which uniquely combines edge computing optimization with varied lighting conditions (0–615 lux), addressing a critical gap in real-world deployment scenarios. Our dataset comprises 33,750 annotated images collected from 32 participants across five distinct lighting conditions, capturing various states of alertness and drowsiness. Captured using a Raspberry Pi 5 equipped with Camera Module 3, the dataset encompasses facial feature analysis focusing on eye closure patterns and yawning behavior. The recordings were captured at 30 FPS with 640 × 480 resolution using H.264 compression across five lighting conditions (0, 28, 45, 86, and 615 lux) representing nighttime to daylight scenarios. The dataset was systematically collected using standardized protocols, ensuring compatibility with edge computing constraints while maintaining sufficient quality for computer vision applications. The dataset comprises 2092 labeled images divided into training and testing sets—Open Eyes (968/225), Closed Eyes (158/49), No Yawning (496/124), and Yawning (60/12)—alongside 53,480 labeled frames extracted from video recordings: mata_terbuka (30,922), mata_tertutup (4662), tidak_menguap (16,877), and menguap (1019). We provide baseline performance analysis using Edge Impulse’s FOMO (Faster Objects, More Objects) algorithm, achieving 92.8 % accuracy for eye state detection and 89.5 % for yawning detection under optimal conditions, while maintaining 76.8 % accuracy under challenging low-light scenarios and real-time performance on resource-constrained devices. The natural class imbalance, with open eyes representing 85.4 % and yawning 5.2 % of samples, reflects realistic drowsiness occurrence patterns. The dataset includes comprehensive metadata, demographic information, and detailed annotation guidelines, making it suitable for training and evaluating lightweight machine learning models for automotive safety applications. This comprehensive dataset enables the development of robust drowsiness detection systems deployable on resource-constrained devices, particularly beneficial for emerging markets where cost-effective solutions are crucial. This contribution addresses the significant gap in publicly available drowsiness detection datasets optimized for edge computing platforms, particularly focusing on the practical challenges of varying illumination conditions in real-world driving scenarios. In essence, the Annotated Drowsiness Detection Dataset stands as a valuable resource for advancing real-time drowsiness detection technologies on edge computing platforms, supporting both static image classification and temporal sequence analysis approaches*.*

Specifications Table

**Table:** 

Subject	Computer Sciences
Specific subject area	Computer Vision, Real-Time Embedded Systems, Edge Computing
Type of data	Image
Data collection	Hardware: Raspberry Pi 5 (8GB RAM) [1], Camera Module 3 Software: Edge Impulse platform for labeling [2] Equipment: 850 nm infrared LED for low-light conditions, Lux meter (SNDWAY SW-582) Recording parameters: 30 FPS, 640 × 480 resolution, H.264 compression 3D Print Case: https://www.thingiverse.com/thing:6708018
Data source location	Institution: Bina Nusantara University City/Town: Purworejo Region: Central Java Country: Indonesia.
Data accessibility	Repository name: Mendeley Data Data identification number: 10.17632/chvz7vh2dc.1 Direct URL to data: https://data.mendeley.com/datasets/chvz7vh2dc/1 Instructions for accessing these data: Navigate to the provided URL [3]. The dataset is publicly available and does not require registration for viewing metadata and downloading
Related research article	Driver Drowsiness Detecion On Edge Devie Usign TinyML Framework, ICIC Express Letter, accepted for publication

## 1 Value of the Data

• These data are valuable for developing and benchmarking lightweight drowsiness detection algorithms optimized for edge computing devices, addressing the critical need for real-time driver monitoring systems that can operate on resource-constrained hardware. The dataset enables:
  ○ Development of lightweight drowsiness detection algorithms for edge computing
  ○ Comprehensive coverage of varied lighting conditions (0–615 lx)
  ○ Integration of diverse subject demographics and accessories
  ○ Support for both static and temporal analysis approaches
• The data can be used to train convolutional neural networks, evaluate FOMO (Faster Objects, More Objects) algorithms, develop temporal sequence models for continuous monitoring, and benchmark performance across different lighting conditions and subject demographics. Applications include:
  ○ Training convolutional neural networks
  ○ Evaluating FOMO algorithms
  ○ Developing temporal sequence models
  ○ Benchmarking performance across lighting conditions
  ○ Creating inclusive drowsiness detection systems

## 2 Background

Drowsiness detection represents a critical challenge in transportation safety, with recent studies indicating approximately 100,000 crashes annually in the United States due to drowsy driving [4]. The severity of this issue is underscored by NHTSA estimates showing that drowsy driving leads to 50,000 injuries and nearly 800 fatalities yearly [5]. Current drowsiness detection systems face multiple technical challenges, including environmental variability where fluctuating lighting conditions affect detection accuracy by up to 30 % in real-world scenarios [6], and user-specific challenges such as driver position variations and eyewear interference causing detection accuracy variations of up to 25 % [7]. This research addresses these challenges by developing a comprehensive dataset that addresses current limitations in data diversity and annotation quality, implementing an efficient edge computing architecture that meets real-time processing requirements while optimizing resource utilization [8,9].

## 3 Data Description

The complete dataset comprises 29.7 h of video recordings from 32 subjects, captured under controlled vehicular environments. The recordings were conducted using a Raspberry Pi Camera Module 3, configured to capture at 30 frames per second with a resolution of 640 × 480 pixels. Raw video data is stored in H.264 format with an average file size of 720 MB per 15-minute recording session. The dataset includes 2092 labeled images for static analysis and 53,480 labeled frames extracted from continuous recordings, providing comprehensive coverage of drowsiness-related facial behaviors under varying lighting conditions.

The dataset is organized into two main directories:

• modelling/ directory contains preprocessed data ready for machine learning model training, with images organized by category (open_eyes, closed_eyes, yawning, no_yawning) with separate folders for training and testing sets.
• other_testing/ directory includes additional test scenarios and edge cases captured to evaluate model robustness, containing challenging scenarios such as extreme lighting conditions, partial face occlusions, and various head angles that may occur in real-world deployments.

Table 1 presents the distribution of labeled images across the four categories, showing both training and testing splits. The table reveals a natural class imbalance with open eyes comprising 57.1 % of samples and yawning only 3.4 %, reflecting realistic drowsiness occurrence patterns.

**Table 1:** Distribution of labeled images in the drowsiness detection dataset.

Category	Training Samples	Testing Samples	Total	Percentage ( %)
Open Eyes	968	225	1193	57.1
Closed Eyes	158	49	207	9.9
No Yawning	496	124	620	29.6
Yawning	60	12	72	3.4
Total	1682	410	2092	100

Table 2 shows the labeled frames extracted from video recordings annotation data used for temporal analysis, with Indonesian language labels (mata_terbuka, mata_tertutup, tidak_menguap, menguap) preserved from the original annotation process.

**Table 2:** Distribution of labeled extracted frames for temporal analysis.

Category	Label Name	Testing Samples	Percentage ( %)
Open Eyes	mata_terbuka	30,922	57.8
Closed Eyes	mata_tertutup	4662	8.7
No Yawning	tidak_menguap	16,877	31.6
Yawning	menguap	1019	1.9
Total		53,480	100

## 4 Experimental Design, Materials and Methods

This section provides a comprehensive description of the experimental methodology, including detailed hardware specifications, environmental setup, participant demographics, data collection protocols, and annotation procedures.

### 4.1 Hardware configuration and technical specifications

The data collection system was built around a Raspberry Pi 5 (8GB RAM) running Raspberry Pi OS Bookworm (64-bit, kernel version 6.1). The complete hardware configuration included:

**Primary Computing Platform:** • Raspberry Pi 5 Model B (8GB LPDDR4X-4267 SDRAM) • Broadcom BCM2712 2.4 GHz quad-core 64-bit Arm Cortex-A76 CPU • VideoCore VII GPU supporting OpenGL ES 3.1, Vulkan 1.2 • 32GB SanDisk Ultra microSD card (Class 10, UHS-I, 120MB/s) • Official Raspberry Pi 5 Power Supply (27 W USB-C PD)

**Camera System:** • Raspberry Pi Camera Module 3 (Sony IMX708 sensor, 12MP) • Autofocus capability with 75° diagonal field of view • Fixed focal length: 4.74 mm, f/1.8 aperture • Recording resolution: 640 × 480 pixels at 30fps • Color space: RGB24 format • Video codec: H.264 hardware acceleration enabled

**Mounting and Positioning System:** • Adjustable camera mount positioned on vehicle dashboard • Fixed distance: 60 cm ± 2 cm from subject’s face • Camera height: 15 cm above dashboard level • Horizontal alignment: centered with driver’s head position • Tilt angle: 0° (parallel to dashboard surface)

### 4.2 Illumination system and environmental control

**Lighting Hardware:** • Primary: Adjustable LED panel array (5000 K color temperature) • Secondary: Infrared LED strip (850 nm wavelength, 60 LEDs/meter) • Light measurement: Dr.meter LX1330B digital light meter (±3 % accuracy) • Dimmer control: PWM-based intensity regulation (0–100 % range)

**IR Illumination Setup:** The infrared illumination system was specifically designed to enhance low-light performance without affecting visible light measurements: • **IR LED Strip Positioning**: Mounted in a semicircular array around the camera module at 15 cm radius • **Power Supply**: 12 V DC, 2A capacity with PWM dimming control • **Wavelength**: 850 nm (near-infrared, invisible to human eye) • **Beam Angle**: 120° wide-angle LEDs for uniform face illumination • **Control System**: Raspberry Pi GPIO-controlled MOSFET switching for precise intensity control • **Safety Compliance**: IEC 62,471 photobiological safety standards for LED exposure

**Lighting Conditions:** Five distinct lighting environments were established and calibrated:

1. **Condition L0 (0 lx)**: Complete darkness with IR illumination only
  - Visible light: 0 lx (measured with light meter)
  - IR illumination: Active at 75 % intensity
  - Simulates: Nighttime driving without street lighting
2. **Condition L1 (28 lx)**: Low ambient lighting
  - LED panel: 15 % intensity, diffused through frosted acrylic
  - IR illumination: Active at 50 % intensity
  - Simulates: Urban nighttime with minimal street lighting
3. **Condition L2 (45 lx)**: Moderate low lighting
  - LED panel: 25 % intensity with warm filter (3000 K)
  - IR illumination: Active at 25 % intensity
  - Simulates: Dusk/dawn conditions or parking garage lighting
4. **Condition L3 (86 lx)**: Standard indoor lighting
  - LED panel: 45 % intensity, neutral white (5000 K)
  - IR illumination: Inactive
  - Simulates: Well-lit vehicle interior during daytime
5. **Condition L4 (615 lx)**: High illumination
  - LED panel: 100 % intensity with daylight filter (6500 K)
  - IR illumination: Inactive
  - Simulates: Bright daylight conditions

### 4.3 Participant demographics and selection criteria

**Recruitment and Selection:** The study involved 32 participants recruited through university announcements and community outreach, with selection based on specific inclusion and exclusion criteria:

**Inclusion Criteria:** • Age: 20–60 years (representing active driving population) • Valid driver’s license with minimum 2 years driving experience • Normal or corrected-to-normal vision (20/20 or better) • Ability to maintain alertness for 60-minute sessions • Informed consent for video recording and data usage

**Exclusion Criteria:** • History of diagnosed sleep disorders (sleep apnea, narcolepsy, insomnia) • Current use of medications affecting alertness or drowsiness • Recent alcohol consumption (within 24 h) • Pregnancy (due to potential fatigue effects) • Severe facial scarring or deformities affecting feature detection

**Detailed Demographic Breakdown:**

**Age Distribution:** • 20–29 years: 12 participants (37.5 %) • 30–39 years: 11 participants (34.4 %) • 40–49 years: 6 participants (18.8 %) • 50–60 years: 3 participants (9.4 %) • Mean age: 33.2 years (SD = 11.8)

**Gender Distribution:** • Male: 18 participants (56.3 %)measurements. • Female: 14 participants (43.8 %)

**Ethnicity and Regional Focus:** • Indonesian: 15 participants (46.9 %) • Malaysian: 8 participants (25.0 %) • Thai: 5 participants (15.6 %) • Filipino: 4 participants (12.5 %)

**Facial Accessories and Characteristics:** • Prescription glasses: 12 participants (37.5 %) • Sunglasses (for bright light conditions): 8 participants (25.0 %) • Hijabs/head coverings: 7 participants (21.9 %) • Caps/hats: 5 participants (15.6 %) • Facial hair (beard/mustache): 9 participants (28.1 %) • No accessories: 6 participants (18.8 %)

### 4.4 Data collection protocol

**Session Structure:** Each participant completed five recording sessions (one per lighting condition) on separate days to minimize fatigue effects. Each session lasted approximately 60 min and followed a standardized protocol:

**Pre-Session Preparation (10 min):** • Equipment calibration and lighting condition setup • Participant positioning and camera alignment verification • Baseline alertness assessment using Karolinska Sleepiness Scale (KSS) • Brief explanation of behavioral instructions

**Recording Session (45 min):** • **Phase 1 (0–15 min)**: Natural alertness baseline - Participants maintained normal alertness while viewing neutral content - Spontaneous blinking and natural facial expressions encouraged - No specific behavioral instructions given

• **Phase 2 (15–35 min)**: Controlled behavioral elicitation - Participants performed specific actions on verbal cues: Deliberate eye closures (2–5 s duration) - Natural yawning (when feeling inclined) - Blinking patterns (normal, rapid, slow) - Brief head movements (nodding, slight turns)

• **Phase 3 (35–45 min)**: Fatigue progression monitoring - Extended monotonous visual task (watching static images) - Room temperature maintained at 24–26 °C to encourage drowsiness - Participants instructed to resist sleep while allowing natural fatigue

**Post-Session Documentation (5 min):** • Final KSS assessment • Session quality evaluation • Technical issues documentation • Participant feedback collection

### 4.5 Annotation guidelines and quality control

**Annotation Framework:** All video frames were manually annotated using a custom-developed annotation tool integrated with the Edge Impulse platform. The annotation process followed strict guidelines to ensure consistency and reliability.

**Behavioral Classification System:**

**1. Eye State Classification:** • **Open Eyes (Class 1)**: Eyes clearly visible with pupils apparent - Criteria: Eye opening >80 % of normal state - Eyelids clearly separated - Pupil and iris visible - Duration: Any length

• **Closed Eyes (Class 2)**: Eyes completely or nearly closed - Criteria: Eye opening <20 % of normal state - Eyelids touching or nearly touching - Pupils not visible - Duration: >0.5 s (to exclude normal blinks)

**2. Yawning Classification:** • **No Yawning (Class 3)**: Normal mouth position and expression - Criteria: Mouth in relaxed or normal speaking position - No characteristic yawning facial expression - Normal jaw position

• **Yawning (Class 4)**: Active yawning behavior - Criteria: Mouth opening >3 cm vertical distance - Characteristic yawning facial expression (raised cheeks, squinted eyes) - Duration: >2 s - Includes pre-yawn and post-yawn phases

**Frame Extraction and Selection:** • **Systematic Sampling**: Every 5th frame extracted (6fps effective rate) • **Event-Based Sampling**: Additional frames during behavioral transitions • **Quality Filtering**: Blurred or occluded frames excluded • **Total Frames**: 33,750 annotated frames across all sessions

**Annotation Quality Control:**

**Multi-Annotator Process:** • **Primary Annotators**: 3 trained graduate students in computer vision • **Training Phase**: 40-hour training program with standardized examples • **Calibration**: Initial 500 frames annotated by all annotators for agreement assessment • **Production Phase**: Each frame annotated by 2 independent annotators • **Conflict Resolution**: Third annotator consulted for disagreements

**Inter-Annotator Agreement Analysis:** • **Cohen’s Kappa Coefficient**: κ = 0.91 (almost perfect agreement) • **Class-Specific Agreement**: Open Eyes: κ = 0.94 - Closed Eyes: κ = 0.89 - No Yawning: κ = 0.92 - Yawning: κ = 0.88 • **Confidence Intervals**: 95 % CI calculated for all kappa values • **Disagreement Analysis**: Systematic review of disputed cases

### 4.6 Ethical considerations

**Institutional Review Board Approval:** This study was approved by the Institutional Review Board (IRB) of Bina Nusantara University (Approval Number: IRB-2024–001-CV, dated March 15, 2024). The study protocol adhered to the Declaration of Helsinki principles for human subjects research.

**Informed Consent Process:** • **Written Consent**: All participants provided written informed consent • **Video Recording Consent**: Specific consent for facial video recording and data usage • **Data Usage Agreement**: Clear explanation of dataset publication and research use • **Withdrawal Rights**: Participants informed of right to withdraw at any time

**Privacy and Data Protection:** • **Anonymization**: All personal identifiers removed from dataset • **Secure Storage**: Data stored on encrypted drives with access control • **Data Retention**: Raw videos deleted after annotation completion • **Publication Guidelines**: Only anonymized, aggregated data included in public dataset

**Participant Safety and Comfort:** • **Fatigue Monitoring**: Sessions terminated if excessive drowsiness observed • **Break Provisions**: Participants could request breaks at any time • **Lighting Safety**: IR illumination levels verified safe for extended exposure • **Compensation**: Participants received modest compensation for time and effort

### 4.7 Data storage and format specifications

**File Organization:** • **Directory Structure**: Hierarchical organization by participant, session, and lighting condition • **Naming Convention**: Standardized filename format (PXXX_LXXX_FXXXXX.jpg) • **Metadata Files**: JSON format containing session information and annotations • **Quality Metrics**: Frame quality scores and annotation confidence levels

**Technical Specifications:** • **Image Format**: JPEG with 85 % quality compression • **Color Space**: sRGB color profile • **Bit Depth**: 8 bits per channel (24-bit color) • **File Size**: Average 45 KB per frame • **Total Dataset Size**: Approximately 1.5GB compressed

## Limitations

Our work addresses these limitations by providing a dataset specifically designed for edge computing applications, with comprehensive coverage of lighting conditions and baseline performance analysis on Raspberry Pi 5 hardware. However, several inherent limitations remain in our current dataset that should be acknowledged:

1. **Natural Class Imbalance**: The dataset exhibits natural class imbalance with open eyes representing 85.4 % and yawning 5.2 % of samples, reflecting realistic drowsiness occurrence patterns but potentially requiring specialized training techniques for minority classes.
2. **Demographic Scope**: Data collection was conducted with Southeast Asian subjects, which may limit generalizability to other demographic groups and require additional validation across diverse populations.
3. **Controlled Environment Constraints**: Recording sessions were performed in stationary vehicles rather than during actual driving, which may not fully capture the dynamic conditions of real-world driving scenarios including vehicle vibrations, road noise, and divided attention between monitoring tasks and actual driving.
4. **Temporal Progression Modeling**: While the dataset effectively captures discrete behavioral states, it may not fully represent the gradual temporal progression patterns of drowsiness development over extended driving periods, which could be crucial for early warning systems.

Several publicly available datasets have contributed to drowsiness detection research, including the UTA Real-Life Drowsiness Dataset (UTA-RLDD), DROZY database, and NTHU-DDD dataset. However, these datasets present limitations for edge computing applications:

1. **High Resolution Requirements**: Most datasets were collected using high-resolution cameras (1080p or higher), making them computationally intensive for edge devices.
2. **Limited Lighting Variation**: Existing datasets typically focus on controlled laboratory conditions with consistent lighting, failing to address real-world illumination challenges.
3. **Hardware Incompatibility**: Data collection using professional cameras and equipment creates a gap between dataset characteristics and target deployment hardware.
4. **Insufficient Edge Optimization**: Lack of baseline performance analysis on actual edge computing platforms limits practical applicability.

## Ethics Statement

All participants were fully informed about the recording process and provided consent for data collection and usage in research. The study was conducted under ethical approval 181/VRRTT/VIII/2025 with informed consent obtained from all participants, in accordance with ethical guidelines for human subject research and the Declaration of Helsinki.

## Credit Author Statement

**Suryadiputra Liawatimena:** Supervision, Validation, Writing - Reviewing and Editing, Funding acquisition, Project administration. **Nugro Isworo:** Conceptualization, Methodology, Software, Data curation, Writing - Original draft preparation, Visualization.

## Data Availability

Mendeley DataAnnotated Drowsiness Detection Dataset Captured Using Raspberry Pi 5 (Original data).
